# The incidence of diabetes among 0–34 year olds in Sweden: new data and better methods

**DOI:** 10.1007/s00125-014-3225-9

**Published:** 2014-04-09

**Authors:** Araz Rawshani, Mona Landin-Olsson, Ann-Marie Svensson, Lennarth Nyström, Hans J. Arnqvist, Jan Bolinder, Soffia Gudbjörnsdottir

**Affiliations:** 1 Nationella Diabetesregistret, Registercentrum VGR, 413 45 Gothenburg, Sweden; 2 Department of Clinical Science, Lund University, Lund, Sweden; 3 Department of Clinical Science, Umeå University, Umeå, Sweden; 4 Department of Clinical and Experimental Medicine, Linköping University, Linköping, Sweden; 5 Department of Endocrinology, Metabolism and Diabetes, Karolinska Institutet, Stockholm, Sweden

**Keywords:** Epidemiology, Incidence, Spring harvest theory, Type 1 diabetes

## Abstract

**Aims/hypothesis:**

We reassessed the validity of previously reported incidence rates for type 1 diabetes in 0–34 year olds in Sweden. We estimated new incidence rates through three nationwide registers.

**Methods:**

We used capture–recapture methods to assess ascertainment in the Diabetes Incidence Study in Sweden (DISS) and estimated incidence rates in the 20–34 year age group for 2007–2009. We examined whether incidence rates in patients aged 34 and younger could be estimated through the Prescribed Drug Register (PDR) via a proxy for diagnosis of type 1 diabetes; men with at least one and women with at least three prescriptions for insulin were included if they had not been given oral glucose-lowering drugs. We scrutinised the proxy by comparing incidence rates in patients aged 14 and younger with the Swedish Childhood Diabetes Register (SCDR), which has 95–99% ascertainment, and by assessing diabetes type among 18–34 year olds in the National Diabetes Register (NDR).

**Results:**

Incidence rates were two to three times higher than previously reported. The absolute number of cases (2007–2009, age 20–34) was 435 in the DISS, 923 in the NDR, 1,217 in the PDR, 1,431 in all three and 1,617 per the capture–recapture method. Ascertainment in the DISS was ~29% for 2007–2009. The proxy diagnosis in the PDR was highly reliable, while the capture–recapture method presumably generated an overestimate.

**Conclusions/interpretation:**

The incidence of type 1 diabetes in patients aged 34 and younger was two to three times higher than previously reported. The PDR can be used to reliably assess incidence rates in this age group.

**Electronic supplementary material:**

The online version of this article (doi:10.1007/s00125-014-3225-9) contains peer-reviewed but unedited supplementary material, which is available to authorised users.

## Introduction

Type 1 diabetes is an epidemiological enigma. It was a rare disease in the early twentieth century. Its incidence began to rise in the 1950s [1]. International multicentre studies initiated in the 1980s included subjects aged 14 and younger. The studies have reported an annual 3% increase in the incidence of the disease. The steepest increase has been noted in the age range 0–4 years, and it appears that onset has shifted to younger age groups [2, 3]. The incidence in Europe is predicted to rise by an alarming 70% between 2005 and 2020 [3].

The explanation for this increase remains elusive. A possible hypothesis is that improved survival has increased the prevalence of susceptible genes. However, the rapid increase in the incidence of the disease and the striking spatiotemporal variations cannot be explained by genetic changes alone [1, 4, 5]. It follows that environmental factors are making a major contribution. Something has changed in our environment, causing more individuals to develop the disease, at a younger age and with less genetic predisposition [6, 7].

It has been postulated that the increase in patients aged 14 and younger represents a left shift in the age of onset and that this increase is mirrored by a corresponding decrease in the rest of the population. The competing theory states that the incidence has risen in people aged 15 and older as well [1]. Epidemiological studies have been contradictory. Two out of three noteworthy studies supporting a left shift in the age of onset, without an increase in cumulative incidence, were Swedish [8–10]. Reports from Finland [11], Italy [12] and the UK [13], however, showed stable or increasing incidence up to the age of 39.

Resolving these contradictions requires the use of standardised registers with adequate age range and geographic coverage, as well as a high level of ascertainment. Such an approach demands initiatives and resources, and failure at any step along the way can lead to erroneous conclusions. Indeed, hypotheses on the spring harvest theory proceed from scarce data for the 15–34 age group. We used three nationwide registers and capture–recapture methods to reassess the validity of previous reports from Sweden and to estimate new incidence rates. We also explored whether incidence could be monitored by means of the Prescribed Drug Register (PDR) alone.

## Methods

### Data sources

Swedish researchers and public authorities manage several nationwide registers that are suitable for our aims. Linking these registers with the personal identification number assigned to each Swede is a straightforward process.

Started in 1996, the National Diabetes Register (NDR) covers patients aged 18 and older [14]. Data provided by trained nurses and physicians include information obtained at both outpatient and primary care clinics. Data are transmitted seamlessly from the clinic to the NDR. Individuals with type 1 diabetes are almost invariably treated at the outpatient clinics of hospitals, more than 90% of which reported to the NDR during the study period. Ninety-five percent of the subjects of the study had been diagnosed with type 1 diabetes on the basis of a clinical assessment. The remaining 5% were being treated with insulin only and had developed the disease at age 30 or younger; these sample characteristics have been validated in 97% of cases [15]. Between 12% and 15% of patients eligible for inclusion were excluded because of missing data for either the year of onset or type of diabetes.

The Diabetes Incidence Study in Sweden (DISS), which began in 1983, includes incident cases of Swedes aged 15–34 years diagnosed at departments of paediatrics, internal medicine and endocrinology [16, 17]. The patients are reported on a data entry form. Each clinic appoints a physician to serve as a contact, provide information about the DISS and make sure that new cases are reported. Classification of the diabetes type is based on a clinical assessment, as well as an analysis of islet cell antibodies since 1998. We included individuals classified as having type 1 diabetes in the DISS. The level of ascertainment in the DISS is uncertain—previous checks have relied on data from 1983 to 1997, including roughly 15% of the at-risk population [8, 18]. Importantly, previous Swedish reports that have contributed substantially to the spring harvest theory are based on a combination of the DISS and the Swedish Childhood Diabetes Register (SCDR).

Started in 1977, the SCDR includes patients aged 14 and younger. The SCDR participates in international multicentre studies and has a level of ascertainment approaching 100% through efficient entry of incident cases, stringent validation procedures and vigorous data collection. Although our study did not use the SCDR, we compared our estimates with its previous reports from 2005 to 2007 [19].

The PDR of the National Board of Health and Welfare contains data on every prescription filled in Sweden since 1 July 2005 [20]. We used a proxy for diagnosis of type 1 diabetes in the age range 0–34 years in the PDR (database available from 1 July 2005 to 31 December 2012). Men receiving at least one prescription and women receiving at least three prescriptions (thereby excluding gestational diabetes) of insulin were classified as having type 1 diabetes if they had never been given oral glucose-lowering drugs. The date of receiving the first prescription was regarded as the onset of the disease. This method was scrutinised by comparing incidence rates in patients aged 14 and younger with available figures from the SCDR in 2005–2007 [19]. We assessed the type of diabetes in patients aged 18–34 via the NDR if it was possible to match them there. Data in the PDR for 2005, 2006 and 2012 were ineligible. The PDR started in mid-2005, and cases were accumulated for 2005 and 2006. The ineligibility of 2012 was due to missing data for 2013 (inclusion of 2013 would affect incidence rates for women since they have to receive at least three prescriptions of insulin). The remaining 6,245 individuals (58% men) were incident cases of type 1 diabetes in 2007–2011.

This analysis included the PDR, DISS and NDR. Incidence rates were calculated separately in each register and collectively by capture–recapture methods.

### Procedures

We calculated incidence rates in the NDR (20–34 year olds for 2006–2011), the DISS (15–34 year olds for 2006–2009) and the PDR (0–34 year olds for 2007–2011) separately.

We used a three-sample capture–recapture procedure to estimate the level of ascertainment in the NDR, the DISS and the PDR [21, 22]. We used a sample of all cases in the 20–34 age group with disease onset in 2009; the three registers overlap for these populations, and the differences in the individual incidence rates were smallest in 2009. Ascertainment for each register was calculated as the actual number of cases divided by the capture–recapture estimate. We also used the capture–recapture method to estimate new incidence rates by age group and sex in 2007–2009 (all three sources were available for this period).

Chapman’s estimators were used for pairwise comparison of registers. Pairwise estimates should be similar if sources are independent [23, 24]. Log-linear models, which allow exploration and incorporation of dependence and heterogeneity, were used to estimate true population size. Our estimated level of ascertainment and incidence rates is based on the best-fitting log-linear model [21, 22]. We also used the sample coverage approach, which is able to estimate population size and dependence (the latter is modelled by the coefficient of covariation [CCV]) [25].

Approved by the Central Ethical Review Board at the University of Gothenburg, the study was conducted in accordance with the principles of the Declaration of Helsinki. Statistical analyses were performed with SAS V.9.3 (SAS Institute, Cary, NC, USA) and R (R Foundation for Statistical Computing).

## Results

In terms of the proxy for diagnosis of type 1 diabetes, 91% of the cases identified in the PDR among the 18–34 age group could be matched in the NDR. Ninety-one per cent of the cases were classified as type 1 diabetes in the NDR. When the analysis was restricted to patients aged 18–30, 94% of cases were classified as type 1 diabetes.

Given the level of ascertainment in 2009 for 20–34 year olds, 151 cases were reported in the DISS, 312 in the NDR, 406 in the PDR and 475 altogether. Chapman’s estimates were 503 (PDR and NDR), 380 (NDR and DISS) and 457 (PDR and DISS). The results suggest positive dependence between the NDR and DISS, since it is lower than other pairwise estimates. Disregarding dependence, the sample coverage approach estimated 504 (95% CI 492, 523) patients in the population, while the log-linear model estimated 520 (95% CI 504, 539). The sample coverage approach indicated a slight dependence between the PDR and DISS (CCV assuming independent sources 0.10; CCV assuming dependent sources 0.20) and no dependence between the NDR and PDR (CCV assuming independent sources 0.00; CCV assuming dependent sources 0.10). The dependence was stronger between the NDR and DISS (CCV assuming independent sources 0.33; CCV assuming dependent sources 0.45). Heterogeneity in capture probabilities was revealed in both log-linear models and the sample coverage approach. Thus, incorporating both dependency and heterogeneity resulted in better-fitting log-linear models. The best-fitting log-linear model, which included the interaction between the NDR and DISS, generated an estimate of 528 (95% CI 508, 554) patients in the population. Thus, the level of ascertainment was 29% in the DISS, 59% in the NDR and 77% in the PDR. The sample coverage approach for dependent sources generated an estimate of 551 (95% CI 524, 594) patients. The level of ascertainment was also calculated for 2007 and 2008; the results were very similar.

**Fig. 1:**
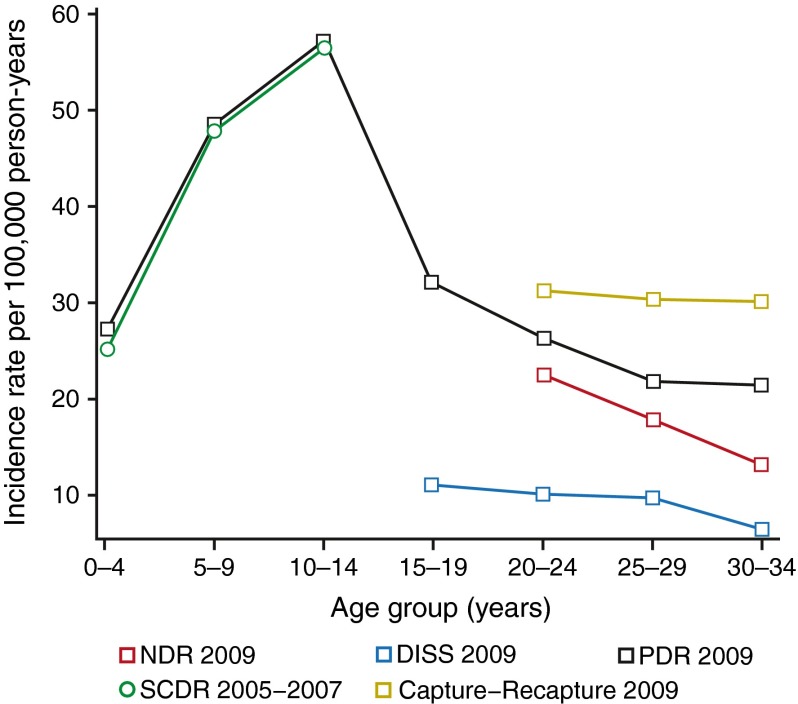
Incidence rates per 100,000 person-years by age group and register in 2009 (men and women). Data from the NDR, DISS, PDR and the capture–recapture method included all three sources. Data from the SCDR (2005–2007) are also presented for comparison purposes; these data came from Berhan et al [19]

Table 1 and Fig. 1 present incidence rates obtained in separate registers and by means of capture–recapture methods using log-linear models (see electronic supplementary material (ESM) for tables and figures by sex). Please refer to the tables and figures for the details of incidence rates. For patients aged 14 and younger, the incidence rates obtained in the PDR were very similar to those reported by the SCDR in 2005–2007 [19]. For the 15–19 age group, we had data from the PDR and DISS only, the results showing that incidence rates are two to three times higher in the PDR than in the DISS. For the 20–34 age group, we compared all three registers separately, as well as their combined capture–recapture estimates (2007–2009). In terms of the separate registers, the DISS had the lowest incidence and the PDR had the highest (generally twice as high). However, the highest incidence rates were obtained by means of the capture–recapture method. The incidence rate in the PDR among 20–24 year olds in 2009 was 26.5 (95% CI 22.3, 30.6) per 100,000 person-years, while the capture–recapture estimate was 31.2 (95% CI 26.7, 35.7) per 100,000 person-years.

**Table 1:** Incidence rates by age group and register (men and women)

Source	Age group	Year					
		2006	2007	2008	2009	2010	2011
NDR							
	20–24	126; 23.6 (19.5, 27.7)	112; 20.4 (16.6, 24.2)	112; 19.7 (16.0, 23.3)	134; 22.6 (18.8, 26.4)	109; 17.6 (14.3, 20.9)	100; 15.6 (12.5, 18.6)
	25–29	106; 19.4 (15.7, 23.1)	100; 18.1 (14.6, 21.7)	109; 19.5 (15.8, 23.2)	101; 17.8 (14.3, 21.3)	94; 16.3 (13.0, 19.6)	91; 15.5 (12.3, 18.6)
	30–34	83; 13.7 (10.7, 16.6)	103; 17.2 (13.9, 20.6)	75; 12.7 (9.8, 15.6)	77; 13.2 (10.3, 16.2)	63; 10.8 (8.2, 13.5)	51; 8.7 (6.3, 11.1)
DISS							
	15–19	96; 15.8 (12.6, 18.9)	100; 15.9 (12.8, 19.0)	81; 12.7 (9.9, 15.4)	71; 11.1 (8.5, 13.7)	N/A	N/A
	20–24	69; 12.9 (9.9, 16.0)	67; 12.2 (9.3, 15.1)	51; 9.0 (6.5, 11.4)	60; 10.1 (7.6, 12.7)	N/A	N/A
	25–29	53; 9.7 (7.1, 12.3)	58; 10.5 (7.8, 13.2)	49; 8.8 (6.3, 11.2)	55; 9.7 (7.1, 12.3)	N/A	N/A
	30–34	51; 8.4 (6.1, 10.7)	39; 6.5 (4.5, 8.6)	20; 3.4 (1.9, 4.9)	36; 6.2 (4.2, 8.2)	N/A	N/A
PDR							
	0–4	N/A	116; 22.4 (18.3, 26.4)	106; 20.0 (16.2, 23.8)	146; 26.9 (22.5, 31.3)	132; 23.8 (19.7, 27.8)	150; 26.6 (22.3, 30.8)
	5–9	N/A	230; 48.7 (42.4, 55.0)	239; 49.5 (43.3, 55.8)	240; 48.4 (42.2, 54.5)	233; 45.7 (39.8, 51.6)	246; 47.0 (41.1, 52.9)
	10–14	N/A	293; 52.8 (46.8, 58.9)	265; 50.1 (44.1, 56.2)	290; 57.2 (50.6, 63.8)	277; 56.3 (49.6, 62.9)	266; 54.6 (48.1, 61.2)
	15–19	N/A	230; 36.6 (31.9, 41.3)	203; 31.8 (27.4, 36.1)	206; 32.2 (27.8, 36.6)	219; 34.8 (30.2, 39.5)	204; 33.7 (29.1, 38.3)
	20–24	N/A	132; 24.0 (19.9, 28.1)	126; 22.1 (18.3, 26.0)	157; 26.5 (22.3, 30.6)	139; 22.5 (18.7, 26.2)	136; 21.2 (17.6, 24.8)
	25–29	N/A	142; 25.8 (21.5, 30.0)	125; 22.4 (18.5, 26.3)	124; 21.9 (18.0, 25.7)	116; 20.1 (16.4, 23.8)	135; 22.9 (19.1, 26.8)
	30–34	N/A	156; 26.1 (22.0, 30.2)	130; 22.1 (18.3, 25.9)	125; 21.5 (17.7, 25.2)	98; 16.9 (13.5, 20.2)	113; 19.4 (15.8, 22.9)
CARE							
	20–24	N/A	177; 32.1 (27.4, 36.9)	174; 30.6 (26.1, 35.2)	185; 31.2 (26.7, 35.7)	N/A	N/A
	25–29	N/A	210; 38.2 (33.0, 43.3)	188; 33.7 (28.9, 38.5)	172; 30.4 (25.8, 34.9)	N/A	N/A
	30–34	N/A	258; 43.2 (37.9, 48.4)	182; 30.8 (26.4, 35.3)	176; 30.2 (25.7, 34.7)	N/A	N/A

Values are absolute number of cases; incidence rates per 100,000 person-years (95% CI)

CARE, capture–recapture estimate using the PDR, the DISS and the NDR; N/A, not applicable

In the PDR, the mean age at diagnosis for women decreased significantly (*p* < 0.001) from 15.6 for 2007, 15.6 for 2008, 14.8 for 2009, 14.3 for 2010 to 14.1 for 2011. The mean age at diagnosis for men was 16.7 for 2007, 16.0 for 2008, 15.8 for 2009, 15.6 for 2010 and 15.9 for 2011, with a non-significant analysis of variance (*p* = 0.060).

## Discussion

Sweden and Finland manage some of the largest registers of diabetes care and research. Given that the two countries also have the highest incidence rate of type 1 diabetes in the world, their reports are important [3]. Two out of three noteworthy studies supporting a left shift in the age of onset without an increase in cumulative incidence are Swedish. These studies were based on the DISS, which includes 15–34 year olds, and the SCDR, which includes patients aged 14 and younger [8, 9]. The third study is from Belgium, a country with rather a low incidence [10]. Reports from Finland for 1992–2001 [11], Italy for 1984–2004 [12] and the UK for 1991–1999 [13], on the other hand, indicate increasing or stable incidence in patients aged 39 and younger.

We compared three nationwide Swedish registers separately and collectively by means of a capture–recapture method in order to reassess the validity of previous reports and estimate new incidence rates. Our analysis showed that the DISS register had a level of ascertainment of ~29% for 2007–2009, which is much too low to ensure reliable epidemiological data. The DISS had better ascertainment earlier; the total number of reported cases has decreased from 367 in 1983, 357 in 1993, 329 in 2003 and 216 in 2009. Our results should seriously call into question previous Swedish reports on the subject. It also suggests that epidemiological evidence supporting the spring harvest theory may have to be discarded. However, it does not negate the theory per se, given that other observations support it, as discussed comprehensively by Gale [1]. The same author proposes that a large multinational study to explore the prevalence of islet autoantibodies in the background population should resolve the issue beyond dispute. The argument is that type 1 diabetes rarely develops in the absence of islet autoantibodies, and their prevalence in the background population should reflect whether initiating factors determine the difference in incidence between populations. Conversely, equal antibody prevalence supports different rates of disease progression. However, previous reports from Sweden have underestimated the incidence rate of type 1 diabetes in the 15–34 age group.

We hypothesised that incidence rates could be monitored solely via a proxy for diagnosis of type 1 diabetes in the PDR. Using the PDR, we found that the incidence rate in the 15–34 age group was two to three times higher than in the DISS. The PDR showed that incidence rates in young people were equal to those in children aged 0–4 years. The proxy for diagnosis was scrutinised and appeared to be reliable. The method’s ascertainment capability was assessed by comparing incidence rates in patients aged 14 and younger with figures from the SCDR (2007–2009), which has an almost 100% level of ascertainment; the results were very similar [19]. We showed that the risk of including other types of diabetes was very small: 91% of cases identified in the PDR for the 18–34 age group could be recaptured in the NDR, where 91% were classified as type 1 diabetes. When only 18–30 year olds were considered, 94% were classified as having type 1 diabetes.

However, incidence rates and population sizes estimated by the capture–recapture method were higher than in the PDR. We note the difference in the nature of the three registers used by our study. All three registers are nationwide. Data entry in the NDR and DISS depends on the active participation of clinics, as well as patient consent. Our analysis showed dependence with regard to entry in the NDR and DISS, perhaps reflecting patterns of clinical practice (i.e. differing proclivities to engage in research and conduct quality assurance projects). However, entry in the PDR is a passive and inevitable consequence of the disease. All individuals with type 1 diabetes must receive insulin, and it is impossible to do so in Sweden without having been entered in the PDR. Thus, regardless of entry in the various registers, all patients will be referred to the PDR, but the PDR does not issue referrals. The PDR includes virtually every Swede with type 1 diabetes. The delay from disease onset to receipt of the first prescription for insulin should be no more than 2–10 days. Moreover, classification of the type of diabetes differs among the three registers. Misclassification leads to inclusion of other types of diabetes, which inflates the estimated population, particularly if the NDR and DISS have low levels of ascertainment. This could explain why incidence rates and population sizes obtained via capture–recapture methods were higher than those in the PDR.

Given the nature of the disease and inevitable entry in the PDR, we believe that our proxy for diagnosis in the PDR (particularly for patients aged 30 and younger) is a reliable and feasible approach to future monitoring. However, the method is not flawless in young adults. Whereas type 1 diabetes in childhood often involves a straightforward diagnosis, autoimmune diabetes in adults is poorly defined [26] despite reports indicating that 25–50% of type 1 diabetes cases are diagnosed in adults [27] and 5–15% of adults diagnosed with type 2 diabetes may actually have type 1 diabetes [28]. The increasing prevalence of obesity in adolescents and young adults blurs the picture further. Thus, the specificity of the PDR could be improved by including islet autoantibodies, perhaps by linking PDR data to research and quality assurance registers by means of such information.

The scientific community needs to establish cost-effective, standardised and reliable methods for monitoring type 1 diabetes—including adolescents and young adults—in large geographic areas. Our study illustrates the challenges and opportunities associated with use of the PDR to monitor the epidemiology of type 1 diabetes.

In conclusion, this study calls into question previous evidence supporting a left shift in age of onset without an increase in cumulative incidence. We report that the incidence of type 1 diabetes is two to three times higher in 15–34 year olds. Our capture–recapture estimates are likely to be inflated because of misclassification of the type of diabetes in the sources we included. We believe that the PDR is probably the gold standard for monitoring the incidence of type 1 diabetes, particularly in patients aged 30 and younger. This method is feasible, reliable and cost-effective.

### Limitations

This study was limited to a relatively short period of time, making it difficult to draw any conclusions about trends. Moreover, we do not present evidence against the spring harvest theory, we simply discard evidence supporting it.

## Electronic supplementary material

Below is the link to the electronic supplementary material.

ESM (PDF 228 kb)

## Abbreviations

CCV: Coefficient of covariation
DISS: Diabetes Incidence Study in Sweden
NDR: National Diabetes Register
PDR: Prescribed Drug Register
SCDR: Swedish Childhood Diabetes Register
