# Supplementary material for: The incidence of diabetes among 0–34 year olds in Sweden: new data and better methods
Source: Diabetologia. 2014 Apr 9;57(7):1375–81. doi: 10.1007/s00125-014-3225-9 (PMC4052006; doi:10.1007/s00125-014-3225-9)
Supplement: Supplementary file 1 — (PDF 228 kb) [file 125_2014_3225_MOESM1_ESM.pdf]

ELECTRONIC SUPPLEMENTARY MATERIAL

INCREASING INCIDENCE OF TYPE 1 DIABETES AMONG 0-34 YEAR OLDS IN SWEDEN

ARAZ RAWSHANI ET AL

On behalf of the Swedish National Diabetes Register (NDR)

CONTENTS

TABLE I: Incidence rates by age group (males)
FIGURE I: Incidence rates by age group (males)

TABLE II: Incidence rates by age group (females)
FIGURE II: Incidence rates by age group (females)

TABLE I: Incidence rates by age group and register (males)

	Age group	Year					
		2006	2007	2008	2009	2010	2011
NDR	20–24	76; 27.8 (21.5, 34.0)	70; 24.9 (19.0, 30.7)	64; 22.0 (16.6, 27.4)	74; 24.4 (18.8, 30.0)	61; 19.3 (14.4, 24.1)	66; 20.1 (15.3, 25.0)
	25–29	59; 21.2 (15.8, 26.6)	74; 26.3 (20.3, 32.3)	61; 21.3 (16.0, 26.7)	56; 19.3 (14.2, 24.3)	52; 17.6 (12.8, 22.3)	56; 18.5 (13.7, 23.4)
	30–34	49; 15.9 (11.4, 20.3)	62; 20.4 (15.3, 25.4)	48; 16.0 (11.5, 20.5)	50; 16.8 (12.2, 21.5)	47; 15.8 (11.3, 20.4)	39; 13.1 (9.0, 17.2)
DISS	15–19	61; 19.5 (14.6, 24.4)	63; 19.5 (14.7, 24.3)	56; 17.0 (12.6, 21.5)	50; 15.2 (11.0, 19.4)	N/A	N/A
	20–24	39; 14.3 (9.8, 18.7)	46; 16.3 (11.6, 21.1)	32; 11.0 (7.2, 14.8)	34; 11.2 (7.4, 15.0)	N/A	N/A
	25–29	36; 12.9 (8.7, 17.2)	48; 17.1 (12.2, 21.9)	29; 10.1 (6.5, 13.8)	30; 10.3 (6.6, 14.0)	N/A	N/A
	30–34	32; 10.4 (6.8, 14.0)	26; 8.5 (5.3, 11.8)	11; 3.7 (1.5, 5.8)	26; 8.7 (5.4, 12.1)	N/A	N/A
PDR	0–4	N/A	59; 22.2 (16.5, 27.8)	58; 21.3 (15.8, 26.7)	81; 29.0 (22.7, 35.4)	64; 22.4 (16.9, 27.9)	81; 27.9 (21.8, 34.0)
	5–9	N/A	111; 45.8 (37.3, 54.3)	126; 50.9 (42.0, 59.8)	133; 52.2 (43.4, 61.1)	121; 46.3 (38.0, 54.5)	128; 47.7 (39.4, 56.0)
	10–14	N/A	168; 59.2 (50.2, 68.1)	149; 55.0 (46.2, 63.9)	167; 64.2 (54.5, 73.9)	151; 59.7 (50.2, 69.3)	141; 56.4 (47.1, 65.7)
	15–19	N/A	148; 45.8 (38.4, 53.2)	127; 38.6 (31.9, 45.4)	140; 42.6 (35.5, 49.6)	137; 42.4 (35.3, 49.5)	132; 42.4 (35.1, 49.6)
	20–24	N/A	80; 28.4 (22.2, 34.7)	70; 24.0 (18.4, 29.7)	96; 31.7 (25.3, 38.0)	80; 25.3 (19.8, 30.8)	92; 28.1 (22.3, 33.8)
	25–29	N/A	96; 34.1 (27.3, 40.9)	76; 26.6 (20.6, 32.6)	71; 24.4 (18.7, 30.1)	68; 23.0 (17.5, 28.4)	77; 25.4 (19.8, 31.1)
	30–34	N/A	81; 26.6 (20.8, 32.4)	70; 23.3 (17.8, 28.7)	83; 27.9 (21.9, 33.9)	61; 20.6 (15.4, 25.7)	78; 26.2 (20.4, 32.0)
CARE[a]	20–24	N/A	97; 34.5 (27.7, 41.4)	92; 31.7 (25.2, 38.1)	106; 35.0 (28.4, 41.7)	N/A	N/A
	25–29	N/A	139; 49.4 (41.2, 57.6)	107; 37.2 (30.2, 44.3)	97; 33.4 (26.7, 40.0)	N/A	N/A
	30–34	N/A	144; 47.4 (39.7, 55.1)	94; 31.3 (24.9, 37.6)	106; 35.5 (28.8, 42.3)	N/A	N/A

Absolute number of cases; incidence rates per 100’000 person-years (95% confidence intervals).
[a]CARE = Capture-recapture estimate using the PDR, the DISS and the NDR.
N/A, not applicable.

FIGURE I: Incidence rates by age group and register (males)

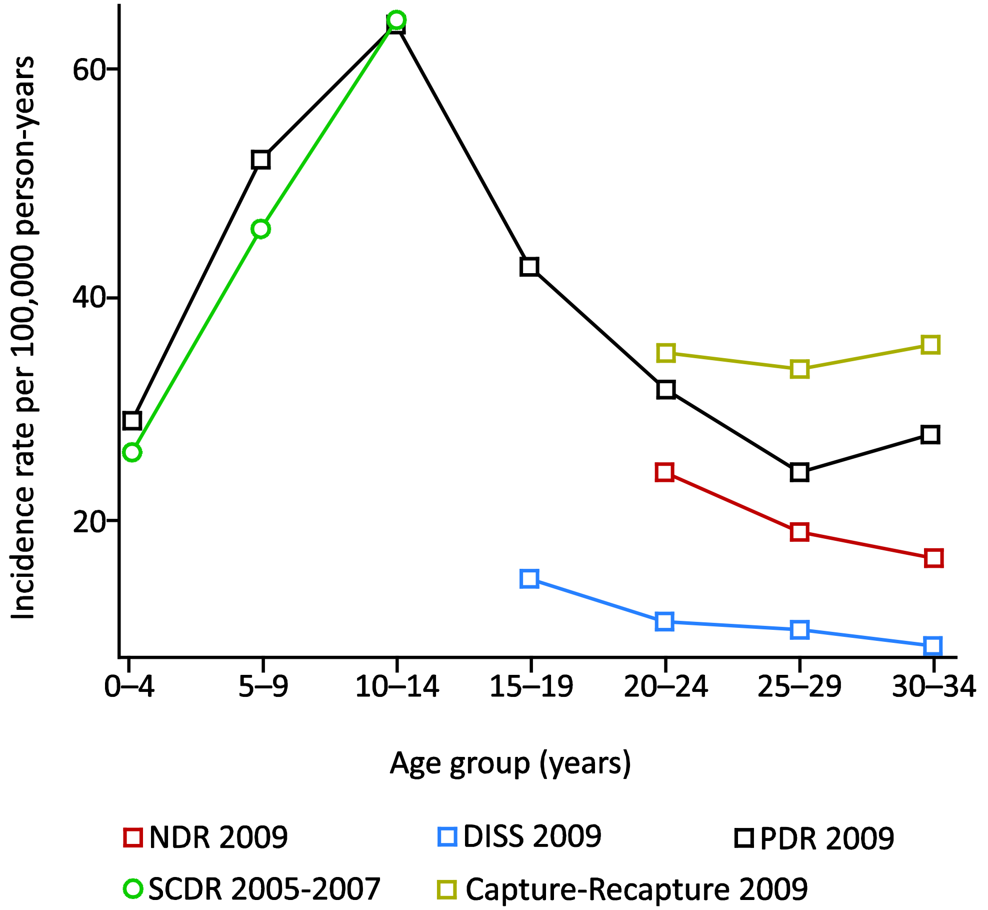

Incidence rates (males) per 100'000 person-years. Capture-Recapture (2007-2009) included the DISS, the NDR and the PDR. Figures from the SCDR (Swedish Childhood Diabetes Register) was obtained from Berhan et al (Diabetes, 2011 Feb;60(2):577-81).

TABLE II: Incidence rates by age group and register (females)

	Age group	Year					
		2006	2007	2008	2009	2010	2011
NDR	20–24	50; 19.2 (13.9, 24.5)	42; 15.7 (10.9, 20.4)	48; 17.3 (12.4, 22.2)	60; 20.7 (15.5, 25.9)	48; 15.9 (11.4, 20.4)	34; 10.8 (7.2, 14.5)
	25–29	47; 17.6 (12.5, 22.6)	26; 9.6 (5.9, 13.3)	48; 17.6 (12.6, 22.6)	45; 16.3 (11.5, 21.0)	42; 15.0 (10.4, 19.5)	35; 12.2 (8.2, 16.3)
	30–34	34; 11.4 (7.6, 15.3)	41; 14.0 (9.7, 18.3)	27; 9.4 (5.8, 12.9)	27; 9.5 (5.9, 13.0)	16; 5.6 (2.9, 8.4)	12; 4.2 (1.8, 6.6)
DISS	15–19	35; 11.8 (7.9, 15.7)	37; 12.1 (8.2, 16.0)	25; 8.1 (4.9, 11.2)	21; 6.8 (3.9, 9.6)	N/A	N/A
	20–24	30; 11.5 (7.4, 15.6)	21; 7.8 (4.5, 11.2)	19; 6.8 (3.8, 9.9)	26; 9.0 (5.5, 12.4)	N/A	N/A
	25–29	17; 6.3 (3.3, 9.4)	10; 3.7 (1.4, 6.0)	20; 7.3 (4.1, 10.5)	25; 9.0 (5.5, 12.6)	N/A	N/A
	30–34	19; 6.4 (3.5, 9.3)	13; 4.4 (2.0, 6.8)	9; 3.1 (1.1, 5.2)	9; 3.2 (1.1, 5.2)	N/A	N/A
PDR	0–4	N/A	57; 22.6 (16.7, 28.4)	48; 18.6 (13.3, 23.8)	65; 24.6 (18.7, 30.6)	68; 25.2 (19.2, 31.2)	69; 25.2 (19.2, 31.1)
	5–9	N/A	119; 51.8 (42.5, 61.1)	113; 48.1 (39.2, 57.0)	107; 44.3 (35.9, 52.7)	112; 45.1 (36.8, 53.5)	118; 46.3 (37.9, 54.6)
	10–14	N/A	125; 46.2 (38.1, 54.3)	116; 45.0 (36.8, 53.2)	123; 49.8 (41.0, 58.6)	126; 52.6 (43.4, 61.8)	125; 52.8 (43.6, 62.1)
	15–19	N/A	82; 26.8 (21.0, 32.7)	76; 24.5 (19.0, 30.0)	66; 21.2 (16.1, 26.3)	82; 26.8 (21.0, 32.7)	72; 24.5 (18.8, 30.1)
	20–24	N/A	52; 19.4 (14.1, 24.7)	56; 20.2 (14.9, 25.4)	61; 21.1 (15.8, 26.3)	59; 19.5 (14.5, 24.5)	44; 14.0 (9.9, 18.2)
	25–29	N/A	46; 17.1 (12.1, 22.0)	49; 18.0 (12.9, 23.0)	53; 19.2 (14.0, 24.3)	48; 17.1 (12.3, 21.9)	58; 20.3 (15.1, 25.5)
	30–34	N/A	75; 25.6 (19.8, 31.4)	60; 20.8 (15.5, 26.0)	42; 14.7 (10.3, 19.2)	37; 13.0 (8.8, 17.2)	35; 12.3 (8.2, 16.3)
CARE[a]	20–24	N/A	83; 31.1 (24.4, 37.7)	83; 29.7 (23.3, 36.1)	80; 27.4 (21.4, 33.5)	N/A	N/A
	25–29	N/A	73; 27.1 (20.9, 33.3)	82; 30.1 (23.6, 36.6)	75; 27.3 (21.1, 33.4)	N/A	N/A
	30–34	N/A	111; 37.9 (30.8, 44.9)	92; 31.8 (25.3, 38.4)	75; 26.2 (20.3, 32.2)	N/A	N/A

Absolute number of cases; incidence rates per 100’000 person-years (95% confidence intervals).
[a]CARE = Capture-recapture estimate using the PDR, the DISS and the NDR.
N/A, not applicable.

FIGURE II: Incidence rates by age group and register (females)

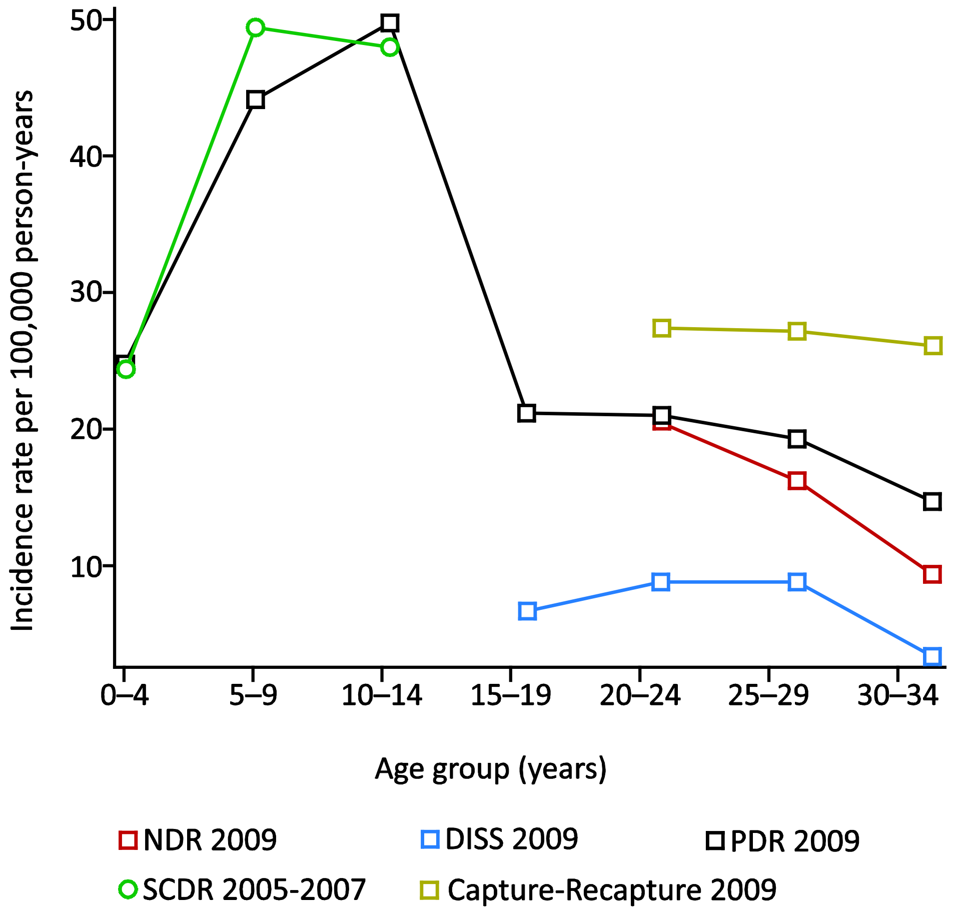

Incidence rates (females) per 100'000 person-years. Capture-Recapture (2007-2009) included the DISS, the NDR and the PDR. Figures from the SCDR (Swedish Childhood Diabetes Register) was obtained from Berhan et al (Diabetes, 2011 Feb;60(2):577-81).
